# The student-teacher framework guided by self-training and consistency regularization for semi-supervised medical image segmentation

**DOI:** 10.1371/journal.pone.0300039

**Published:** 2024-04-22

**Authors:** Boliang Li, Yaming Xu, Yan Wang, Luxiu Li, Bo Zhang

**Affiliations:** 1 Department of control science and engineering, Harbin Institute of Technology, Harbin, Heilongjiang, China; 2 Faculty of Robot Science and Engineering, Northeastern University, Shenyang, Liaoning, China; 3 Sergeant schools of Army Academy of Armored Forces, Changchun, Jilin, China; University of California Los Angeles, UNITED STATES

## Abstract

Due to the high suitability of semi-supervised learning for medical image segmentation, a plethora of valuable research has been conducted and has achieved noteworthy success in this field. However, many approaches tend to confine their focus to a singular semi-supervised framework, thereby overlooking the potential enhancements in segmentation performance offered by integrating several frameworks. In this paper, we propose a novel semi-supervised framework named Pesudo-Label Mean Teacher (PLMT), which synergizes the self-training pipeline with pseudo-labeling and consistency regularization techniques. In particular, we integrate the student-teacher structure with consistency loss into the self-training pipeline to facilitate a mutually beneficial enhancement between the two methods. This structure not only generates remarkably accurate pseudo-labels for the self-training pipeline but also furnishes additional pseudo-label supervision for the student-teacher framework. Moreover, to explore the impact of different semi-supervised losses on the segmentation performance of the PLMT framework, we introduce adaptive loss weights. The PLMT could dynamically adjust the weights of different semi-supervised losses during the training process. Extension experiments on three public datasets demonstrate that our framework achieves the best performance and outperforms the other five semi-supervised methods. The PLMT is an initial exploration of the framework that melds the self-training pipeline with consistency regularization and offers a comparatively innovative perspective in semi-supervised image segmentation.

## Introduction

The segmentation of medical images is a crucial part of the clinical analysis to aid experts in the diagnosis of diseases and the formulation of treatment plans [1]. Deep learning methods have demonstrated significant achievements in medical image segmentation recently [2–4]. However, these approaches rely heavily on annotated data, but the acquisition of labels is a complex and time-consuming process, which substantially encumbers the prospective evolution of deep learning methods in this domain [5]. Semi-supervised learning [6] is highly suitable for medical image segmentation tasks since it can effectively extract information from large amounts of unlabelled images. Therefore, excellent approaches to semi-supervised learning are increasingly emerging.

In various semi-supervised learning methods [7–10], approaches based on self-training and consistency regularization are widely employed due to their simplicity and efficacy. The crucial of self-training is pseudo-labels. This approach first creates pseudo-labels for unlabeled images, then composes an extra training dataset of pseudo-labels and unlabeled images, and finally forces the segmentation model to learn effective information from unlabeled images by pseudo-label supervised loss. Consequently, the precision of pseudo-labels is essential for realizing the best performance in self-training. In the consistency regularization domain, the student-teacher structure is one of the most widely used structures, for example, the mean teacher [9]. It furnishes identical inputs to both student and teacher models but adds additional noise to the inputs of the student model, and supervises the student model using the outputs of the teacher model, thereby enforcing output consistency between the two models and actualizing the low-density separation between different classes in semi-supervised methods.

However, many researchers have focused on developing entirely novel semi-supervised methods or enhancing single existing methods, neglecting the potential benefits derivable from combining several semi-supervised approaches. In this study, we present a novel semi-supervised method called Pseudo Label Mean Teacher (PLMT). It combines the self-training process and the consistency regularisation method. In the PLMT framework, the student-teacher structure based on consistency regularization can yield precise pseudo-labels, whereas the self-training pipeline provides additional pseudo-label supervision for the student-teacher structure. In essence, we amalgamate the two most prevalently employed semi-supervised methods to engender a mutually reinforcing impact.

Furthermore, since the PLMT framework includes pseudo-labeled and consistency losses, the weights of different semi-supervised losses represent the preference of the PLMT framework. It is necessary to consider the impact of different weights of the semi-supervised loss function. In response to this point, we introduce adaptive loss weights, which allow PLMT to dynamically adjust the weights to the optimal values for different tasks during training and thereby achieve the best segmentation accuracy.

An approach similar to ours is the UPC framework [11]. It also leverages both pseudo-labels and consistency regularization. Nevertheless, it diverges from our technique in that it directly utilizes the outputs of the teacher model as pseudo-labels. This strategy could compromise the accuracy of the pseudo-labels, accumulating errors and degrading performance. In contrast, our approach incorporates the student-teacher architecture within the self-training pipeline, thereby facilitating the generation of more precise pseudo-labels. Overall, our main contributions to this paper are summarized as follows:

- We introduce a novel semi-supervised medical image segmentation framework named PLMT, which integrates consistency regularization with the self-training pipeline.
- By adaptively adjusting the weights of the two kinds of unsupervised losses, the PLMT framework can take full advantage of the benefits of both pseudo-label and consistency regularization.
- Experimental results from three datasets demonstrate that our framework can effectively extract task-relevant information from unlabeled samples and outperforms the other five semi-supervised methods.

## Related works

### Consistency regularization

Consistency regularization is one of the most widely applied methods for semi-supervised learning. The basic principle is that the network presents a consistent output for noisy samples. In other words, tiny perturbations should not alter the classification results of the network for the same inputs.

There are many consistency regularization approaches have been developed. For instance, the temporal ensemble [12] method employs a self-ensembling strategy, enforcing consistency in predictions between two augmentations and the network predictions across previous epochs of the same sample. Tarvainen *et al*. [9] proposed the student-teacher structure and explored the prediction consistency of models with different parameters. Miyato *et al*. [13] introduced a virtual adversarial training method that employs adversarial training to establish the consistency constraint between the outputs of unlabeled samples and those with adversarial noise. Additionally, various studies [14–17] have also proposed other effective consistency methods. In this paper, we utilize the student-teacher structure as the implementation mechanism of the consistency regularization in the PLMT framework.

### Self training

Self-training [10] is also known as pseudo-labeling, which essentially means training a model with little labeled data and generating pseudo-labels for the unlabeled data. Recently, it has been increasingly attracted attention and widely used in deep learning. Bai *et al*. [18] introduced a method namely semiFCN that performs self-training for medical image segmentation by amalgamating labeled and unlabeled data during the training process. Yang *et al*. [19] presented the ST++ method for semi-supervised semantic segmentation by employing strong data augmentation on unlabeled data and adjusting the order of usage on the unlabeled data for the reliability of pseudo-labels. Zou *et al*. [20] improved the quality of the pseudo-labels by fusing pixel-level and image-level pseudo-labels and strong data augmentation. Different from the above methods, we merge the student-teacher structure into the self-training stream to yield more precise pseudo-labels.

### Semi-supervised medical image segmentation

Since semi-supervised methods can alleviate the challenge of labeled data, several methods have been applied to medical image segmentation recently. For example, Yu *et al*. [21] amalgamated the student-teacher structure and uncertainty to execute the left atrium segmentation task. Shi *et al*. [22] introduced an uncertainty estimation semi-supervised method designed to capture the inconsistent prediction across multiple cost-sensitive settings to diminish prediction uncertainty. Luo *et al*. [23] explored the dual-task consistency between the segmentation predictions and geometry-aware level-set regression through a dual-task network. Wu *et al*. [24] proposed MC-Net+, which has multiple decoder outputs, for semi-supervised medical image segmentation by establishing consistency restrictions among the outputs of multiple decoders. Similarly, Luo *et al*. [25] utilized a pyramid prediction network, learning from the unlabeled data by encouraging multiple scales to yield consistent predictions. Furthermore, other novel semi-supervised approaches [26–29] have also demonstrated excellent performance in specific medical image segmentation tasks. However, the aforementioned methods seldom concentrate on the relationship between consistency regularization and self-training. In contrast, the PLMT framework integrates the student-teacher structure into the self-training pipeline, facilitating the extraction of additional valuable representations from unlabeled data.

## Method

### Problem definition

**Fig 1:**
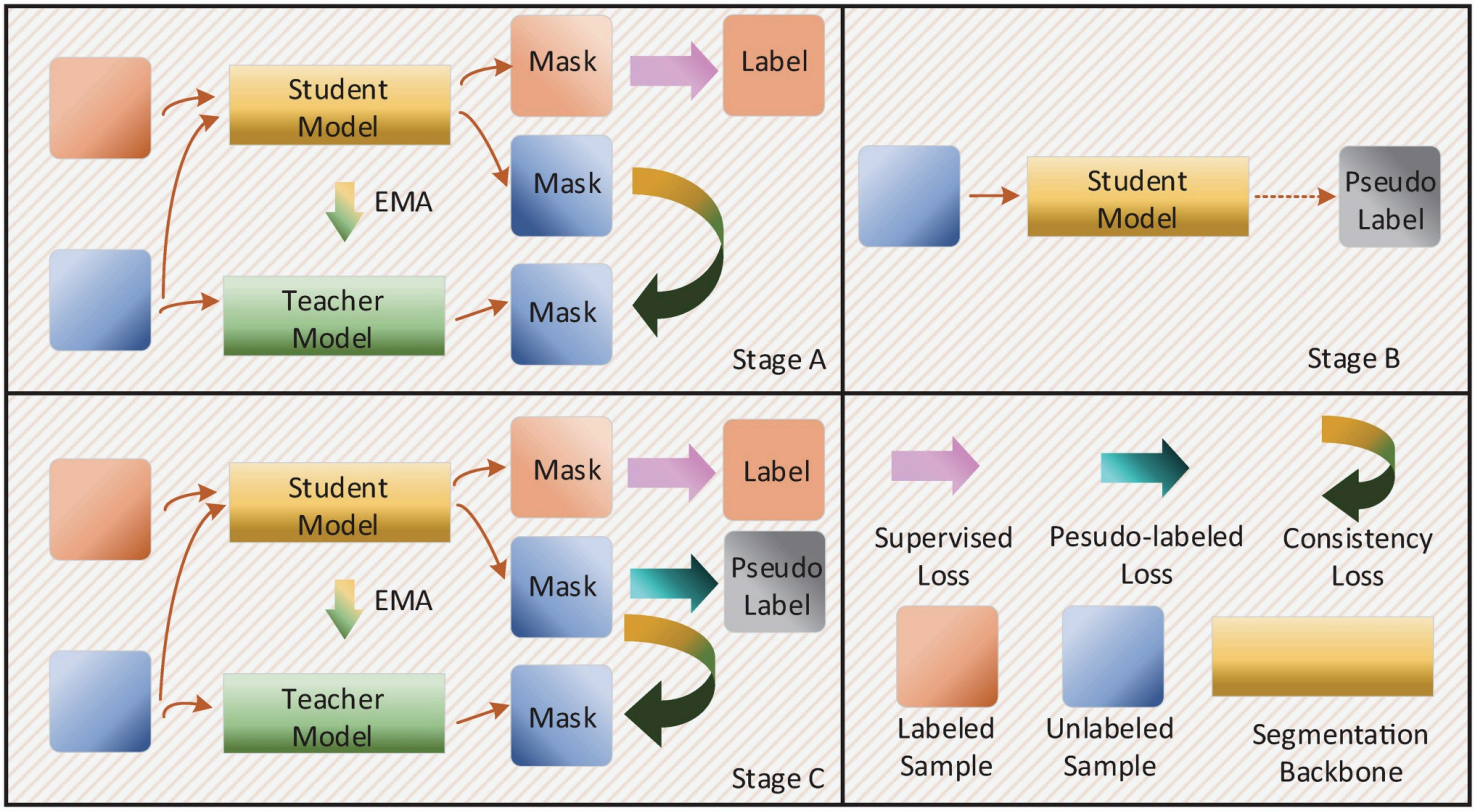
Overview of the proposed PLMT framework.

In this section, we detail the proposed PLMT framework, as illustrated in Fig 1. Before describing our method, we introduce the formula representation of our dataset and network. Due to the rarity of medical image annotation, we target to train the model using a small number of images with annotation and a large number of images without labels to improve the segmentation accuracy on the test dataset. The labeld images $(x_{i}^{l},y_{i}^{l})\in D^{L}\left(1\leq i\leq M\right)$ and unlabeld images $(x_{j}^{u})\in D^{U}\left(1\leq j\leq N\right)$ jointly constitute the training dataset, where $y_{i}^{l}$ refers to the ground truth and *N* ≫ *M*. Acquiring as many task-relevant and efficient representations as possible from unlabelled data is the most critical problem confronted by every semi-supervised medical image segmentation method.

As illustrated in Fig 1, the primary architecture employed in the PLMT is a student-teacher structure, where the parameters of the teacher model are updated by the student model using the exponential moving average (EMA) during the training process. To facilitate description, we donate teacher and student networks by $f(\theta_{•}^{T})$ and $f(\theta_{•}^{S})$. *f*(θ_*_) refers to the network with parameters used for producing the pseudo-labels. The training sample *x*_*i*_ is fed into *f*(*θ*) to obtain the probability output *p*_*i*_. Similarly, during the generation of pseudo-labels, the unlabeled sample $x_{j}^{u}$ is fed into *f*(θ_*_) to yield $p_{j}^{u}$, and generates the corresponding one-hot pseudo-label $y_{j}^{p}$.

### Framework and pipeline

From a general perspective, the PLMT is an end-to-end semi-supervised framework, but in detail, It comprises three primary stages, similar to the self-training workflow. These stages are: (1) training the baseline network to determine its optimal parameters, (2) leveraging the segmentation model with the optimal parameters to produce pseudo-labels, and (3) re-training the segmentation network from scratch while incorporating the pseudo-label supervised loss. For convenience of description, we denote these stages as Stage A, Stage B, and Stage C, as depicted in Fig 1.

During Stage A of self-training, the model can solely be trained to utilize a small amount of labeled data and cannot utilize the information from numerous unlabelled samples. In contrast, in Stage A of PLMT, we employ the Mean Teacher structure, which can fully exploit the vast amount of unlabeled data, optimizing segmentation model parameters and producing precise pseudo-labels, which are essential for achieving outstanding outcomes of the self-training approach.

Stage B of the PLMT aligns with the pseudo-label generation in the self-training approach. In other words, the segmentation model attained from stage A is used to procure pseudo-labels for unlabeled samples. Additional techniques, such as setting the pseudo-label confidence threshold, are not employed to streamline the process.

In Stage C of the PLMT framework, the segmentation network is trained using a Teacher Student structure, integrating pseudo-labels and consistency regularization. Contrasting with the conventional Mean Teacher structure, PLMT provides additional supervision loss from the pseudo-labels. Furthermore, relative to the self-training method, PLMT supplies consistency regularization loss and more precise pseudo-labels, facilitating the extraction of effective representations from unlabeled samples.

**Algorithm 1** The training pipeline of the PLMT framework

**Require**: labeled samples: $b_{l}=\left(x_{i}^{l},y_{i}^{l}\right)\in D^{L}$, unlabeled samples: $b_{u}=\left(x_{j}^{u}\right)\in D^{U}$

**Require**: student and teacher model parameters in Stage A: $f(\theta_{A}^{S})$ and $f(\theta_{A}^{T})$

**Require**: student and teacher model parameters in Stage C: $f(\theta_{C}^{S})$ and $f(\theta_{C}^{T})$

**Require**: maximum iterations: *iter*_*max*

**Require**: semi-supervised loss weight: λ_*A*_ and λ_*C*_

**Require**: trainable parameters: *α*, *β* and temperature factor: *K*

**Ensure**: optimized parameters of the student model in Stage A: $\theta_{A}^{*}$

**Ensure**: segmentation network parameters: $\theta_{C}^{*}$

 ########### Stage A ###########

1: count ← 0

2: **while** count < *iter*_*max* **do**

3:  **for** *b*_*A*_ = *b*_*l*_ + *b*_*u*_ **do**

4:   $L_{sup}^{A}\leftarrow l_{ce}(f\left(x_{i}^{l},\theta_{A}^{S}\right)$, $y_{i}^{l})$

5:   $L_{con}^{A}\leftarrow l_{mse}(f\left(x_{j}^{u},\theta_{A}^{S}\right)$, $f(x_{j}^{u},\theta_{A}^{T}))$

6:   $L_{total}^{A}\leftarrow L_{sup}^{A}+\lambda_{A}\times L_{con}^{A}$

7:   Updating $\theta_{A}^{S}$ by optimizer and updating $f(\theta_{A}^{T})$ by $f(\theta_{A}^{S})$

8:   count ← count+1

9:  **end for**

10: **end while**

 ########### Stage B ###########

11: **for** $b_{u}=\left(x_{j}^{u}\right)$ **in** *D*^*U*^ **do**

12:  $y_{j}^{p}\leftarrow f\left(x_{j}^{u},\theta_{A}^{*}\right)$

13:  $b_{p}=\left(x_{j}^{u},y_{j}^{p}\right)\in D^{U}\leftarrow x_{j}^{u},y_{j}^{p}$

14: **end for**

 ########### Stage C ###########

15: count ← 0

16: **while** count < *iter*_*max* **do**

17:  **for** *b*_*C*_ = *b*_*l*_ + *b*_*p*_ **do**

18:   $L_{sup}^{C}\leftarrow l_{ce}(f\left(x_{i}^{l},\theta_{C}^{S}\right)$, $y_{i}^{l})$

19:   $L_{con}^{C}\leftarrow l_{mse}(f\left(x_{j}^{u},\theta_{C}^{S}\right)$, $f(x_{j}^{u},\theta_{C}^{T}))$

20:   $L_{pse}^{C}\leftarrow l_{ce}(f\left(x_{j}^{u},\theta_{C}^{S}\right)$, $y_{j}^{p})$

21:   $L_{total}^{C}\leftarrow L_{sup}^{C}+\lambda_{C}\times \left(\alpha \times K\times L_{con}^{C}+\beta \times L_{pse}^{C}\right)$

22:   Updating $\theta_{C}^{S}$ by optimizer and updating $f(\theta_{C}^{T})$ by $f(\theta_{C}^{S})$

23:   count ← count+1

24:  **end for**

25: **end while**

26: **return** $\theta_{C}^{*}$

### Loss function

In this subsection, we introduce the loss function in the PLMT framework. Overall, the PLMT optimizes the backbone model in Stage A and Stage C, while Stage B is applied to produce pseudo-labels that do not require a loss function. $L_{total}^{A}$ and $L_{total}^{C}$ are applied to formulate the loss functions required in Stage A and Stage C respectively. The optimization of Stage A resembles that of Mean Teacher. Therefore, The $L_{total}^{A}$ can be described as:

$$\begin{array}{c} L_{total}^{A}=L_{sup}^{A}+\lambda_{A}*L_{con}^{A} \end{array}$$ (1)

where λ_*A*_ is the trade-off weight of supervision and consistency loss.

$L_{sup}^{A}$ employs the standard cross-entropy function to calculate the supervision loss between the labeled samples and corresponding ground truth, which is written as:

$$\begin{array}{c} L_{sup}^{A}=\frac{1}{\left|D^{L}\right|}\sum_{x_{i}^{l}\in D^{L}}l_{ce}\left(f\left(x_{i}^{l},\theta_{A}^{S}\right),y_{i}^{l}\right) \end{array}$$ (2)

where *l*_*ce*_ is the cross-entropy loss function. $L_{con}^{A}$ denotes the consistency loss between the outputs of the teacher and student models using unlabeled samples. In this study, we employ the mean squared error function to compute this loss and the $L_{con}^{A}$ can be written as:

$$\begin{array}{c} L_{con}^{A}=\frac{1}{\left|D^{U}\right|}\sum_{x_{J}\in D^{U}}l_{mse}\left(f\left(x_{u},\theta_{A}^{S}\right),f\left(x_{u},\theta_{A}^{T}\right)\right) \end{array}$$ (3)

where *l*_*mse*_ refers to the mean squared error loss function.

Fig 1 indicates that, compared to Stage A, there is an extra pseudo-label supervision loss introduced into the optimization of Stage C. Hence the $L_{total}^{C}$ is formulated as follows.

$$\begin{array}{c} L_{total}^{C}=L_{sup}^{C}+\lambda_{C}*\left(\alpha *K*L_{con}^{C}+\beta *L_{pse}^{C}\right) \end{array}$$ (4)

In the $L_{total}^{C}$, *α* and *β* are the trainable parameters and respectively indicate the weights of the different unsupervised losses in Stage C, where *α* + *β* = 1 and *α*, *β* > 0. Due to the significant magnitude difference between values the $L_{sup}^{C}$ and $L_{con}^{C}$, we introduce a temperature factor K to bridge this gap, ensuring the PLMT framework does not overfit the smaller loss function during training. This measure ensures the intended purpose of multiple semi-supervised losses by avoiding excessive weight allocation to smaller loss values. The loss functions of $L_{sup}^{C},L_{con}^{C}$ are the same as the $L_{sup}^{A},L_{con}^{A}$. The $L_{pse}^{C}$ refers to the pseudo-label supervised loss of pseudo labels and the outputs of the student model in unlabeled samples. It also adopts the cross-entropy function and is written as:

$$\begin{array}{c} L_{pse}^{C}=\frac{1}{\left|D^{U}\right|}\sum_{x_{j}^{u}\in D^{U}}l_{ce}\left(f\left(x_{j}^{u},\theta_{C}^{S}\right),y_{j}^{p}\right) \end{array}$$ (5)

In a word, the PLMT framework enables to yield of more precise pseudo-labels using the student-teacher model. Additionally, the novel introduced pseudo-label supervision loss augments the performance of this student-teacher architecture. Algo 1 provides an overview of the proposed PLMT approach.

## Experiments

### Datasets and pre-processing

We evaluated our approach in three public datasets, which are ACDC, LA, and Spleen Datasets.

The ACDC Dataset is a public benchmark dataset of the 2017 Automated Cardiac Diagnosis Challenge [30]. It contains 100 labeled MR samples in total and includes annotations for three classes: left ventricle(LV), right ventricle(RV), and myocardium(MYO). For fair training and inference, 80 subjects are allocated to the training set and the remaining 20 to the testing set.

The LA dataset is the benchmark dataset for the 2018 Atrial Segmentation Challenge [31], containing 100 gadolinium-enhanced MR imaging scans for training, with a resolution of 0.625 × 0.625 × 0.625 mm. Since the testing set on LA does not include public labels, following [21, 24], we use 80 samples as the training set, the rest 20 samples are for testing.

The Spleen dataset is one of the ten tasks of the Medical Segmentation Decathlon Challenge [32]. It is collected from patients who are receiving chemotherapy treatments for liver metastases and acquired in the Memorial Sloan Kettering Cancer Center. The dataset consists of 61 CT scans in total but only 41 have expert annotations. Following [33], 33 samples compose the training set and the remaining 8 samples are used as the testing set.

Table 1 describes the division of the samples of the three datasets in detail, with * referring to labeled samples and Δ to unlabeled samples. Due to varying image sizes in three original datasets, we resize all the 3D scans into 256 × 256 2D slices. Afterward, we performed 2D rotation and flip operations across the three datasets for data augmentation and normalized the samples to zero mean and unit variance.

**Table 1:** The split of labeled and unlabeled samples in the training and test datasets.

		Dataset					
		LA		Spleen		ACDC	
Samples		*	Δ	*	Δ	*	Δ
	10%	492	4216	84	764	176	1516
Train set	20%	983	3810	172	676	342	1350
	All	4753	0	848	0	1692	0
Test set		1194		203		210	

### Implementation details

In this paper, our method implementation utilizes the PyTorch framework, executed on an Intel(R) i7 13700k CPU and an NVIDIA 4090 GPU. During the optimization stage, we employ the SGD optimizer with a weight decay of 0.001 and momentum of 0.9, training for 36,000 iterations. An initial learning rate of 0.1 is adopted, with the “poly” strategy dictating the learning rate decay. The batch size is set at 24, the size of supervised and unsupervised samples at 12 each. The total semi-supervised loss weight λ_*A*_ and λ_*C*_ are set to 0.1. The temperature factor K is set to 1000. Following [9, 21], we apply a time-dependent Gaussian warming-up function $\lambda^{d}\left(t\right)=e^{{\left(-5\left(1-\frac{t}{t_{max}}\right)\right)}^{2}}$ to balance the supervised and unsupervised losses, where *t* represents the current iteration count and *t*_*max*_ denotes the maximum iterations.

In addition, we employ a 2D UNet with initial channels of 16 and four downsampling and upsampling modules as the segmentation backbone network. Mean Teacher [9], Self-training [10], Entropy minimization [34], DCT [35] and UAMT [21] are adopted as the comparison methods.

We employ four widely used metrics to evaluate the segmentation performance of all methods, including the Dice similarity coefficient (Dice), Jaccard Index (Jaccard), 95% Hausdorff Distance (HD95), and Average Surface Distance (ASD). Specifically, Dice and Jaccard measure the similarity between the segmentation output and the ground truth. ASD and HD95 capture the boundary differences between the output and the label.

## Results

### Performance on the LA dataset

We present the quantitative results of the LA segmentation task in Table 2. This shows the performance of our proposed method and other five comparative methods, alongside the results of a U-Net model trained with 10%, 20%, and all labeled samples as the reference. Table 2 indicates that the PLMT framework outperforms the other five semi-supervised methods across all evaluation metrics. Specifically, compared with the UNet model without any semi-supervised methods, the Dice coefficient of the PLMT increased by 5.06% and 2.57% when trained with only 10% and 20% of the labeled data, respectively. Compared to the best results obtained by other semi-supervised methods, PLMT shows an improvement of 2.14% and 1.44% in the Dice coefficient. Furthermore, when trained with only 20% of the labeled data, the PLMT framework shows a marginal difference of only 0.92% in the Dice coefficient compared to the results obtained from the UNet model with fully labeled data. It demonstrates that the PLMT framework could effectively leverage unlabeled data to extract more efficient representation and significantly enhance performance over other semi-supervised methods.

**Table 2:** Quantitative comparison results on the LA dataset. Best results are in bold and suboptimal results are in underlined. * and ** indicate p ≤ 0.05 and p ≤ 0.02 from two-sided paired t-test when comparing the PLMT with other methods, respectively.

		Metrics			
Method	Sample	Dice(%)	Jaccard(%)	HD95(p)	ASD(p)
FullSupervision	100%	92.11**	85.42**	3.90**	1.14**
UNet	10%	85.56**	75.36**	9.47**	2.90**
MT	10%	88.48**	79.61**	8.06**	2.32**
DCT	10%	87.65**	78.19**	12.11**	3.52**
EM	10%	87.37**	77.82**	8.23**	2.49**
UAMT	10%	87.16**	77.48**	12.34**	3.68**
Self-Training	10%	87.61**	78.15**	8.99**	2.64**
PLMT(Ours)	10%	**90.62**	**82.95**	**6.63**	**1.93**
UNet	20%	88.62**	79.73**	8.03**	2.50**
MT	20%	89.75**	81.52**	6.82*	2.06*
DCT	20%	89.55**	81.21**	8.04**	2.47**
EM	20%	89.20**	80.62**	7.87**	2.39**
UAMT	20%	89.39**	80.93**	7.84**	2.36**
Self-Training	20%	89.68**	81.38**	7.67**	2.29**
PLMT(Ours)	20%	**91.19**	**83.88**	**6.24**	**1.81**

**Fig 2:**
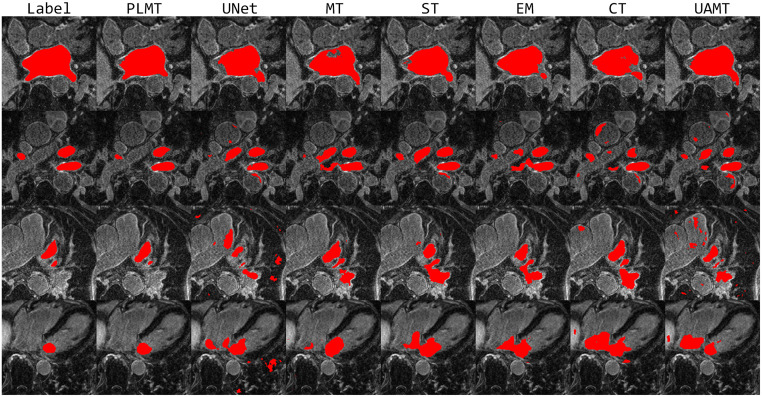
Visual comparison examples on the LA dataset.

To intuitively express the excellent segmentation performance of the PLMT method, we also provide several visualized examples of our framework and other comparison methods in Fig 2. The red portions indicate the segmentation masks resulting from different methods, and the “label” is derived from the corresponding labels of the samples. Compared with other semi-supervised methods, the segmentation masks produced by the PLMT exhibit a closer alignment with the ground truths. It shows that the PLMT could efficiently separate the regions of interest.

### Performance on the Spleen dataset

**Fig 3:**
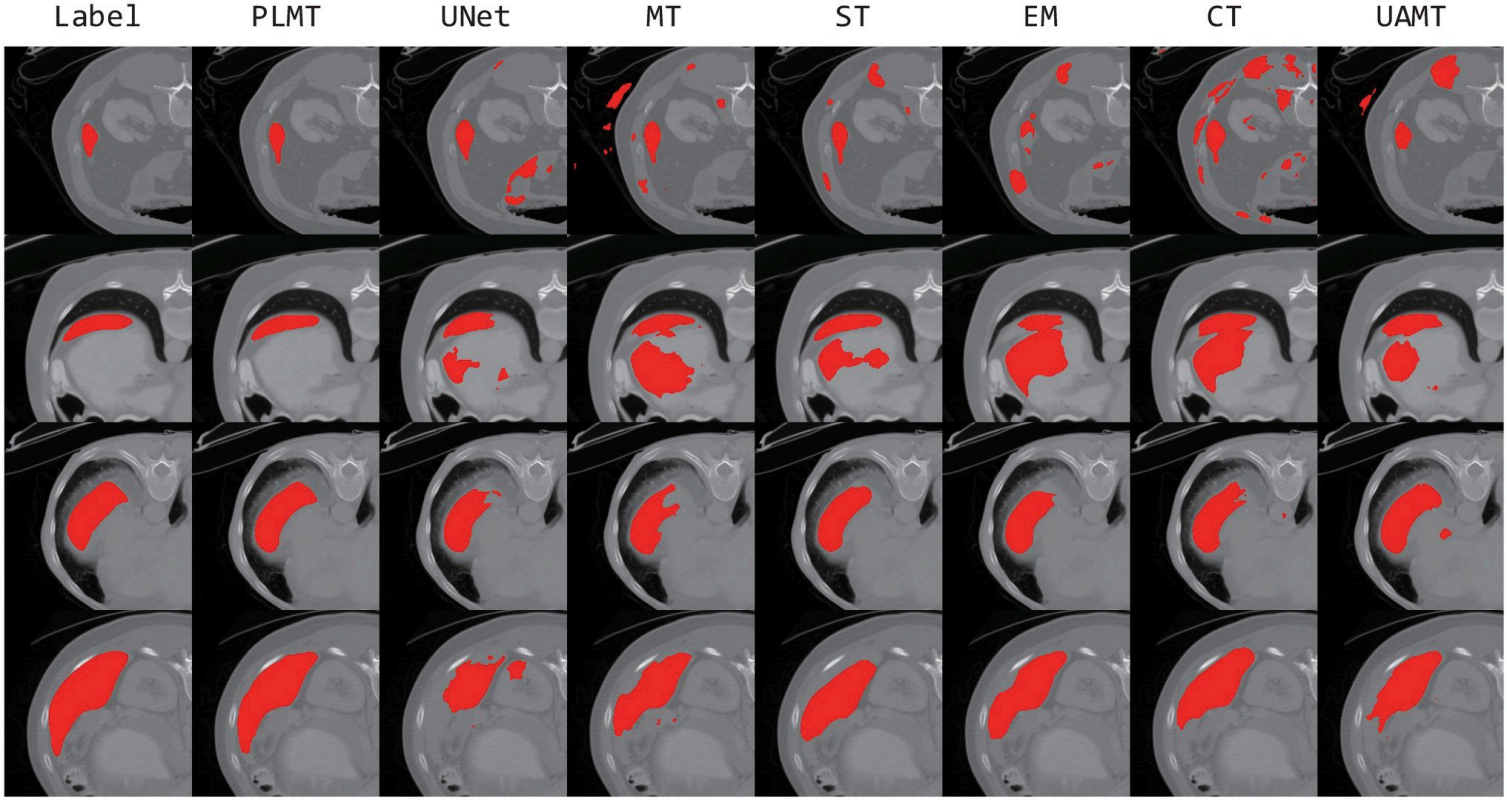
Visual comparison examples on the Spleen dataset.

Similar to the evaluation on the LA dataset, Fig 3 and Table 3 show the corresponding results and visual segmentation examples of the PLMT framework and other comparative methods on the Spleen dataset. It demonstrates that: (1) Relative to the other five semi-supervised methods, our model outperforms in all evaluation metrics, although the ASD is marginally inferior to the self-training framework trained with 10% labeled data. (2) By efficiently leveraging representations from unlabeled data, our model delivers a Dice score improvement of 3.96% and 3.77% over the supervised UNet model trained with 10% and 20% labeled samples, respectively. Compared to the best results obtained by other semi-supervised methods, PLMT shows an improvement of 0.93%(92.88%, DCT with 10% labeled data) and 1.76%(93.81%, DCT with 20% labeled data) in the Dice coefficient. (3) Fig 3 depicts that compared with other segmentation masks, the masks yielded by PLMT enable clear recognition of the target region and exclude erroneous predictions.

**Table 3:** Quantitative comparison results on the Spleen dataset. Best results are in bold and suboptimal results are in underlined. * and ** indicate p ≤ 0.05 and p ≤ 0.02 from two-sided paired t-test when comparing the PLMT with other methods, respectively.

		Metrics			
Method	Samples	Dice(%)	Jaccard(%)	HD95(p)	ASD(p)
FullSupervision	100%	96.10**	92.55**	1.13**	0.30**
UNet	10%	89.85**	82.56**	2.40**	0.67**
MT	10%	91.19**	84.55**	1.83**	0.84**
DCT	10%	92.88**	86.95**	3.37**	1.49**
EM	10%	92.10**	85.75**	3.10**	0.72**
UAMT	10%	91.97**	85.78**	1.96*	1.37**
Self-Training	10%	90.50**	83.83**	2.85**	**0.38**
PLMT(Ours)	10%	**93.81**	**88.58**	**1.52**	0.39
UNet	20%	91.80**	85.52**	2.43**	0.46
MT	20%	93.50**	88.26**	1.81**	0.80*
DCT	20%	93.81**	88.58**	1.52**	0.40
EM	20%	93.68**	88.44**	1.45*	0.50
UAMT	20%	93.22**	87.92**	1.70**	1.10**
Self-Training	20%	93.09**	87.45**	1.81**	0.37
PLMT(Ours)	20%	**95.57**	**91.59**	**1.18**	**0.36**

### Performance on the ACDC dataset

**Fig 4:**
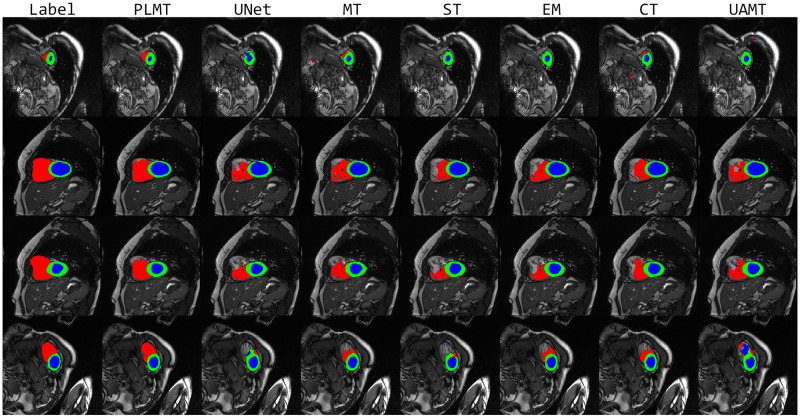
Visual comparison examples on the ACDC dataset.

Different from the LA and Spleen binary classification datasets, ACDC is a multi-classification dataset that includes the right ventricle(RV), myocardium(MYO), and left ventricle(LV) components. Table 4 and Fig 4 show the quantitative results and visualization segmentation examples of the PLMT approach and other methods on the ACDC dataset. It can be seen from Table 4 that the PLMT framework obtains the best performance in most of the evaluation metrics. In 10% labeled sample results, the PLMT achieves a Dice gain of 4.75%, 2.80%, and 2.72% than the UNet without any semi-supervised method in RV, MYO, and LV, respectively. And in 20% labeled sample results, the PLMT achieves a Dice gain of 3.91%, 3.35%, and 2.06%, respectively. In all three categories, the PLMT also achieves the highest Dice score compared to the other five semi-supervised methods. It demonstrates that the approach of combining consistency regularization and self-training indeed yields superior segmentation performance than a single semi-supervised method.

**Table 4:** Quantitative comparison results on the ACDC dataset. Best results are in bold and suboptimal results are in underlined. * and ** indicate p ≤ 0.05 and p ≤ 0.02 from two-sided paired t-test when comparing the PLMT with other methods, respectively.

		Metrics											
		RV				MYO				LV			
Method	Samples	Dice(%)	Jaccard(%)	HD95(p)	ASD(p)	Dice(%)	Jaccard(%)	HD95(p)	ASD(p)	Dice(%)	Jaccard(%)	HD95(p)	ASD(p)
FullSupervision	100%	94.30**	89.29**	1.02**	0.17**	90.98**	83.55**	1.00**	0.24**	95.99**	92.40**	1.01**	0.16**
UNet	10%	84.45**	75.05**	2.31**	0.62**	84.44**	73.40**	1.38	0.73**	90.54**	83.60**	5.25**	1.51**
MT	10%	88.61**	80.38**	1.74*	0.48*	86.43**	76.36**	1.41	**0.38**	93.11*	87.48	**1.80**	**0.41**
DCT	10%	87.64**	78.84**	3.29**	1.04**	85.84**	75.57**	2.11**	0.61	92.44**	86.40**	3.42	1.04*
EM	10%	87.82**	79.29**	2.25**	0.69**	86.13**	75.92**	4.68**	0.97**	91.78**	85.60**	5.76**	1.83**
UAMT	10%	88.35**	79.85**	1.99**	0.56**	86.26**	76.09**	2.05**	0.62	91.43**	85.20**	5.09*	1.43**
Self-Training	10%	86.61**	77.74**	1.74*	0.40	85.68**	75.30**	1.95*	0.47	91.90**	85.76**	3.97	1.09*
PLMT(Ours)	10%	**89.20**	**81.19**	**1.31**	**0.36**	**87.24**	**77.60**	**1.34**	0.57	**93.26**	**87.66**	4.76	0.92
UNet	20%	87.61**	78.91**	2.01**	0.51**	85.29**	74.72**	7.70**	1.75**	92.17**	86.01**	9.04**	2.33**
MT	20%	89.17**	81.07**	1.64*	0.49**	87.27**	77.68**	1.13	0.60	93.55**	88.13**	4.09**	0.92*
DCT	20%	89.33**	81.45**	1.42	0.39*	87.09**	77.40**	7.07**	1.32**	93.24**	87.76**	9.99**	2.14**
EM	20%	88.99**	80.97**	1.54*	0.39*	87.73*	78.40**	**1.10**	0.35	93.52*	88.15**	**1.12**	0.38
UAMT	20%	89.15**	81.20**	1.53*	0.44**	87.57**	78.14**	1.18	**0.31**	93.66*	88.35**	1.17	**0.37**
Self-Training	20%	88.23**	80.01**	1.58*	0.40*	87.54**	78.10**	3.04**	0.74*	93.67*	88.38**	4.08**	0.74*
PLMT(Ours)	20%	**91.52**	**84.55**	**1.30**	**0.21**	**88.64**	**79.76**	1.12	0.39	**94.23**	**89.39**	2.02	0.64

In Fig 4, the red, green, and blue portions indicate the segmentation parts of the right ventricle, myocardium, and left ventricle, respectively. These visual examples show that compared with the segmentation results of other methods, our segmentation maps are very fitted to the ground truths, particularly for the segmentation of the right ventricle, and the mask of the PLMT is significantly better than the results of other semi-supervised methods. Furthermore, the PLMT framework is significantly more precise than other methods in terms of detecting ambiguous boundaries and complex regions.

In a word, based on the results of three datasets, our PLMT framework demonstrates superior performance than the other five semi-supervised methods for medical image segmentation. It should be noted that to purely validate the efficacy of the combination of consistency regularization and self-training pipeline, we do not employ strong data augmentation in the PLMT approach, even though injecting strong data augmentation into the input samples has the potential to improve the performance of semi-supervised segmentation methods, such as those employed in [19, 36].

### Ablation studies

The PLMT framework is an approach that integrates the student-teacher structure into the self-training process with two semi-supervised losses and adaptive loss weights. In addition, the temperature factor K is introduced to bridge the magnitude gap between different semi-supervised loss values. Therefore, in the ablation experiments, we focus on verifying the effectiveness of the PLMT structure and the temperature factor K.

### Effect of the PLMT structure

Since the PLMT structure is the combination of the Mean Teacher and self-training methods, we have demonstrated that the proposed PLMT has superior performance over the single Mean Teacher or self-training by quantitative results on three datasets in Tables 2–4 of the comparison experiments. Therefore, in the ablation experiment, we show more visualization examples to illustrate the PLMT has superior segmentation performance.

**Fig 5:**
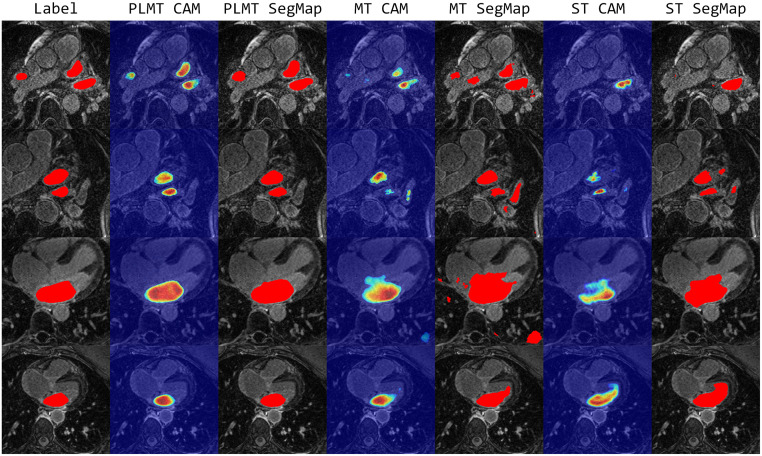
Visual comparison examples on the LA dataset in ablation study.

**Fig 6:**
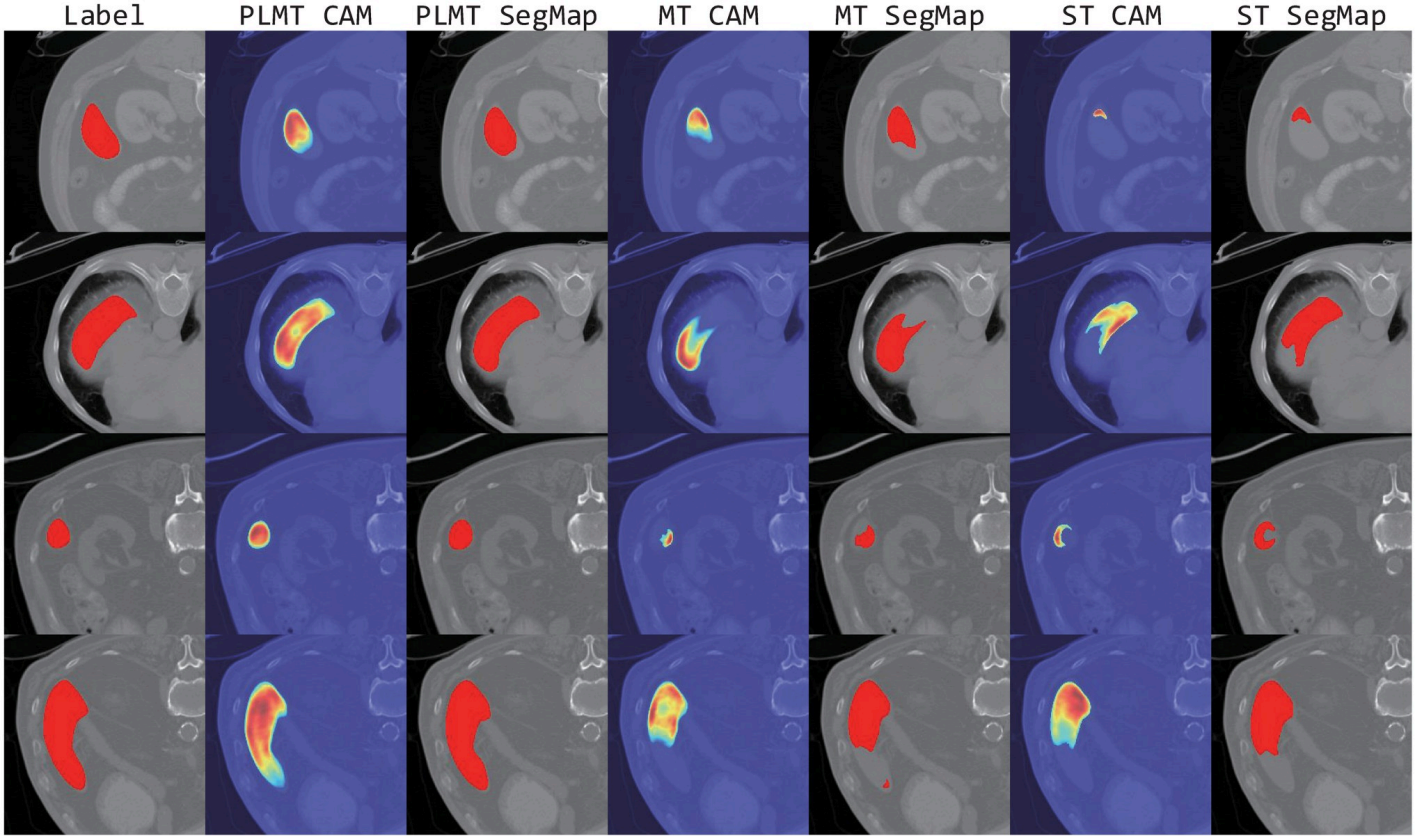
Visual comparison examples on the Spleen dataset in ablation study.

**Fig 7:**
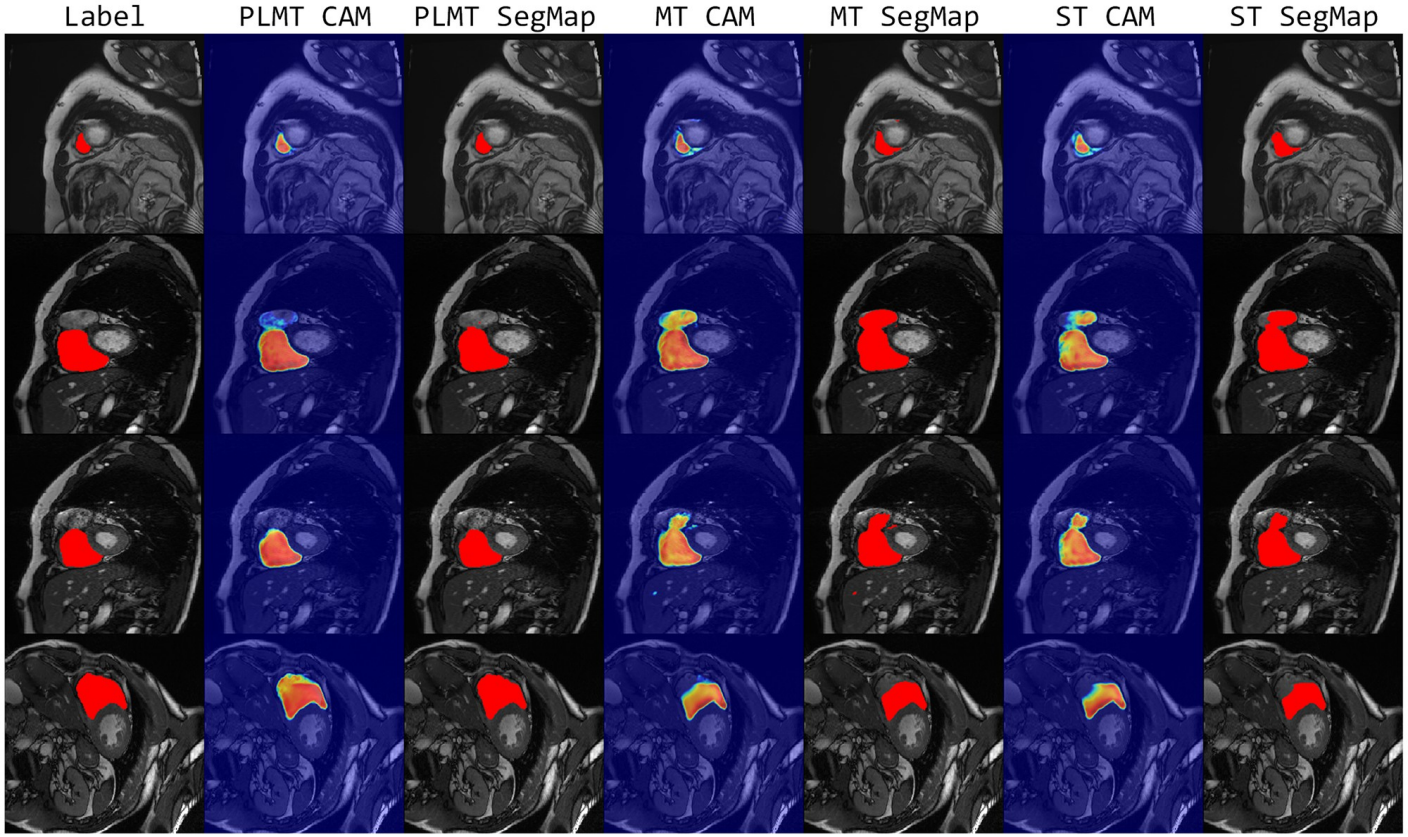
Visual comparison examples on the ACDC dataset in ablation study.

Figs 5–7 show the visual samples of PLMT versus MT and self-training on the three datasets, where “Label” refers to the ground truth corresponding to the sample, “SgeMap” and “CAM” refer to the segmentation maps and corresponding gradient localization maps produced by different semi-supervised methods. As can be seen from Fig 4 the segmentation challenge in the ACDC dataset is primarily in the right ventricle denoted in red portion. Thus we only focus on the right ventricle part for the comparison in Fig 7. From the above figures, we can see that the gradient localization maps resulting from PLMT are more accurate and the segmentation maps are better matched to the labels compared to the single Mean Teacher or self-training methods. It demonstrates that the PLMT framework which integrates two semi-supervised methods is both more accurate and generalizable than a single semi-supervised method.

### Effect of the temperature factor K

The ablation study about the temperature factor K is performed on the LA dataset by using 10% labeled samples, to primarily demonstrate the effectiveness of the value of K and the weights of different unsupervised losses(see Eq 4). Table 5 shows the quantitative results of the ablation study, in which *α* refers to the weight of the consistency loss, *β* refers to the weight of the pseudo loss, the bolded parts indicate the best results and the underlined parts indicate the suboptimal results.

**Table 5:** Quantitative results of the temperature factor K and the adaptive weights *α* and *β* of different unsupervised losses on the LA dataset. Best results are in bold and suboptimal results are in underlined.

			Metrics			
K	*α*	*β*	Dice(%)	Jaccard(%)	HD95(p)	ASD(p)
0	0.292	0.708	86.97	77.20	9.84	3.03
1	0.316	0.684	89.19	80.63	7.28	2.07
500	0.612	0.388	90.29	82.42	6.96	2.01
550	0.617	0.383	90.43	82.65	6.77	2.01
600	0.641	0.359	90.61	82.93	6.48	1.87
650	0.637	0.363	90.48	82.70	7.24	2.16
700	0.684	0.316	90.44	82.64	7.02	2.13
750	0.701	0.299	90.09	82.12	7.51	2.20
800	0.706	0.294	90.62	82.94	6.19	1.82
850	0.720	0.280	90.45	82.67	6.80	1.96
900	0.736	0.264	90.61	82.94	6.34	1.85
950	0.749	0.251	**90.78**	**83.20**	**6.03**	1.76
1000	0.759	0.241	90.62	82.95	6.63	1.93
1050	0.745	0.255	90.74	83.13	6.20	**1.74**
1100	0.780	0.220	90.65	82.99	6.82	1.97
1150	0.754	0.246	90.62	82.94	6.18	1.83
1200	0.767	0.233	90.64	82.97	7.00	2.03

As we can observe from Table 5, there is only pseudo-label loss in the PLMT framework when K = 0, which has the same pipeline as the self-training, but since the PLMT remains with smaller weights for the pseudo-labeling loss, it is unable to take full advantage of the pseudo-labeling loss. Therefore, the performance of the PLMT is inferior compared to the f the corresponding self-training method in Table 2 (Dice score: 87.61% → 86.97%). When K = 1, due to the relatively small value of the consistency loss, the PLMT framework blindly increases the weight of the consistency loss to minimize the overall loss, resulting in the overfitting problem. When K is approximately 1000, the framework effectively bridges the magnitude gap between pseudo-label loss and consistency loss. In this setting, PLMT efficiently leverages the strengths of both semi-supervised losses, resulting in superior segmentation performance. The ablation study demonstrates that fine-tuning the value of the temperature factor K in the PLMT framework further improves the model performance in medical image segmentation tasks.

## Discussion

In the medical image analysis domain, it is expensive and time-consuming to obtain a lot of precisely labeled images. Semi-supervised learning methods can decrease the reliance on labeled data and reduce the cost and time of data preparation. Furthermore, for some rare diseases where it is difficult to obtain enough labeled data, semi-supervised methods could better utilize the small amount of labeled data for more effective research. However, traditional semi-supervised learning methods usually focus only on certain perspectives, such as consistency regularisation or pseudo-labeling. To design a more accurate and robust semi-supervised method, we propose the PLMT framework. Unlike other semi-supervised methods, the PLMT framework integrates the student-teacher structure into the self-training pipeline and combines pseudo-labeling with the consistency regularization method to achieve much more precise segmentation performance. In particular, in the PLMT framework, we utilize the teacher-student structure to obtain more accurate pseudo-labels in Stage A. At Stage C, we establish the teacher-student structure with consistency loss and pseudo-label loss. To better trade off the contribution of two semi-supervised losses for different segmentation tasks, we used adaptive loss weights for different semi-supervised losses, and PLMT could adaptively adjust the weights of different semi-supervised losses, which could achieve more accurate segmentation performance with limited labels. In addition, we introduce the temperature factor K to eliminate the gap between the values of different semi-supervised losses to avoid the risk of overfitting in the PLMT framework.

To validate the performance of the PLMT framework, we evaluate it on three different medical image segmentation tasks to demonstrate its effectiveness and robustness. The comparison results in Tables 2–4 show that the PLMT achieves the best results compared to the other five semi-supervised segmentation methods. In addition, the visual examples in Figs 5–7 also show that the PLMT can achieve more accurate segmentation of lesions or regions of interest with limited labels. From the results in Table 5, it can be observed that the temperature factor K could effectively avoid the overfitting risk arising from the adaptive semi-supervised loss weights in the PLMT framework.

Overall, PLMT is a framework for medical image segmentation that incorporates two semi-supervised methods, which achieves a significant improvement in segmentation performance over single semi-supervised methods such as consistency regularisation or pseudo-label. The PLMT framework demonstrates that incorporating multiple semi-supervised methods from different perspectives can improve the performance of the segmentation backbone from different perspectives. In other words, the PLMT framework illustrates that combining multiple semi-supervised methods can improve the accuracy and robustness of the segmentation model more than a single semi-supervised method. It should be noted that when combining multiple semi-supervised losses, the different semi-supervised loss values must be adjusted to the same magnitude by the temperature factor K to avoid overfitting the framework. In future work, we aim to investigate the framework that can integrate further semi-supervised methods to improve the accuracy and generalization of the medical image segmentation model and to reduce the dependence of segmentation models on labeled data.

Traditional semi-supervised methods due to the training of the segmentation model in both Stage A and Stage C. In future work, the use of more efficient methods to generate more accurate pseudo-labels can further improve the performance of the PLMT framework. In future work, adopting more efficient methods to generate more accurate pseudo-labels can further improve the performance of the PLMT framework.

## Conclusion

In this study, we introduce a novel and efficacious semi-supervised learning framework named PLMT, for medical image segmentation. By synergistically integrating self-training with the Mean Teacher structure, our method outperforms these two standalone semi-supervised learning approaches. Additionally, our method allows for the adaptive adjustment of the loss weights between the consistency and pseudo-label to further optimizer segmentation performance, especially under constraints of limited labeled samples. Extension experiments demonstrate our framework has achieved superior performance compared with the other two methods on three medical datasets. While this research represents an initial exploration into the confluence of self-training and consistency regularization, future work will incorporate diverse strategies to enhance the efficacy of semi-supervised methods in medical image segmentation.

## Supporting information

S1 Fig The setup of different semi-supervised methods. (a) refers to the setting of the mean teacher, (b) refers to the setting of the self-training, and (c) refers to the setting of the proposed PLMT. Since the PLMT framework is the combination of Mean Teacher and self-training methods, the settings in the PLMT framework are the same as in the mean teacher and self-training methods.
(TIF)
