# Supplementary figures and images for: The student-teacher framework guided by self-training and consistency regularization for semi-supervised medical image segmentation
Source: PLoS One. 2024 Apr 22;19(4):e0300039. doi: 10.1371/journal.pone.0300039 (PMC11034649; doi:10.1371/journal.pone.0300039)

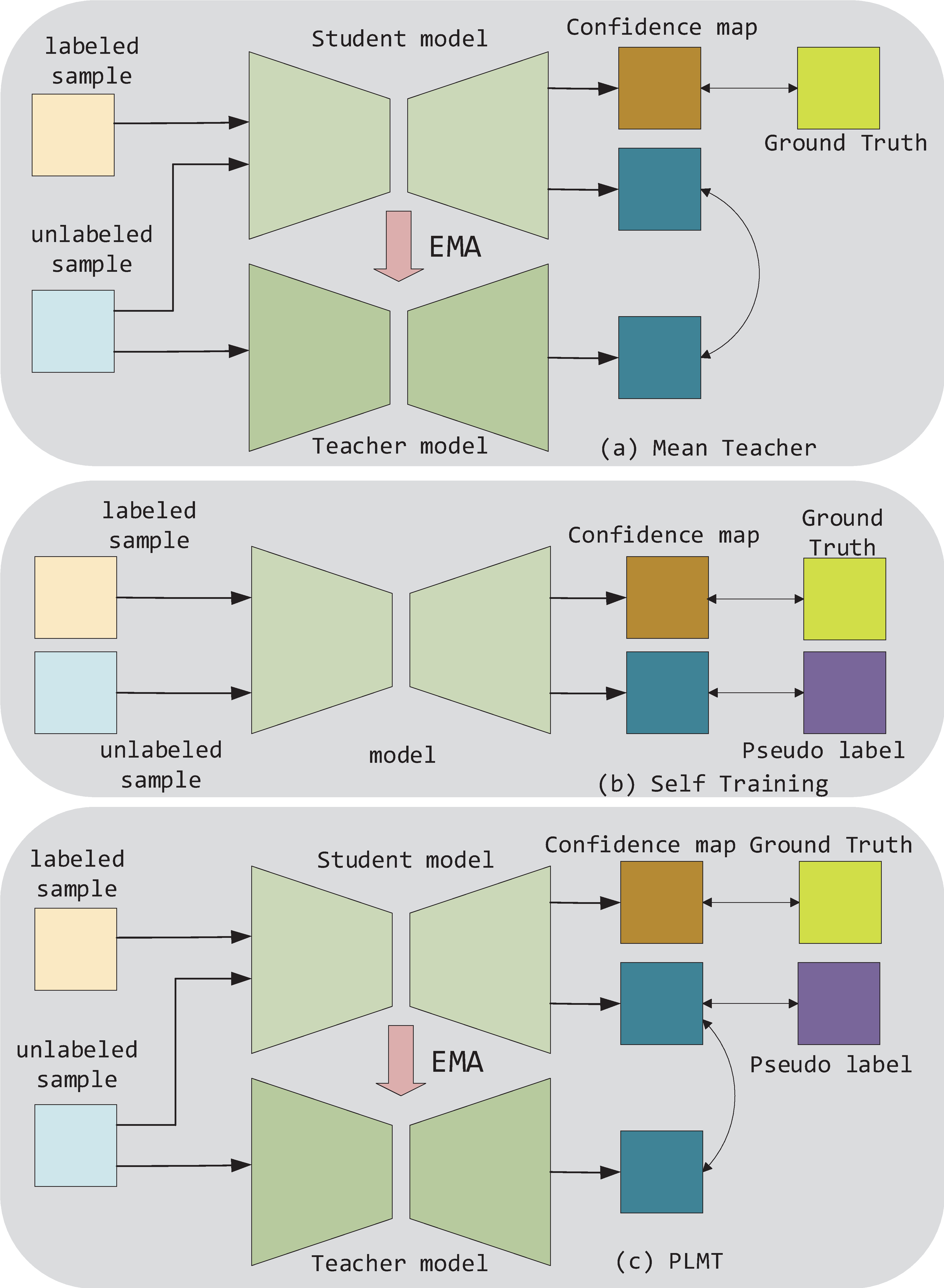

Supplement: S1 Fig — (a) refers to the setting of the mean teacher, (b) refers to the setting of the self-training, and (c) refers to the setting of the proposed PLMT. Since the PLMT framework is the combination of Mean Teacher and self-training methods, the settings in the PLMT framework are the same as in the mean teacher and self-training methods. (TIF) [file pone.0300039.s001.tif]
